# The obstetric and gynecological service providers’ and recipients’ perception and experience of the quality of obstetric triage services during the COVID-19 pandemic in Iran

**DOI:** 10.1186/s12884-023-05351-9

**Published:** 2023-03-01

**Authors:** Hamideh Yazdimoghaddam, Sayyed Majid Sadrzadeh, Fatemeh Zahra Karimi

**Affiliations:** 1 Department of Operating Room, Faculty of Paramedics, Leishmaniasis Research Center, Sabzevar University of Medical Sciences, Sabzevar, Iran; 2 Department of Emergency Medicine, Faculty of Medicine, Mashhad University of Medical Sciences, Mashhad, Iran; 3 Reproductive Health, Nursing and Midwifery Care Research Center, Mashhad University of Medical Sciences, Mashhad, Iran; 4 Department of Midwifery, School of Nursing and Midwifery, Mashhad University of Medical Sciences, Mashhad, Iran

**Keywords:** Obstetric triage, COVID-19, Outbreak, Pandemic, Qualitative study

## Abstract

**Background:**

Enhancing the quality of obstetric triage services requires a clear perception of the current situations and problems, this issue gained more importance during the COVID-19 pandemic. The purpose of this study was to explore the obstetric and gynecological service providers’ and recipients’ perception and experience of the quality of obstetric triage services during the COVID-19 pandemic.

**Methods:**

This research was a qualitative study carried out using conventional content analysis. Participants were selected through purposive sampling, and data collection was conducted using in-depth semi-structured interviews. Data were analyzed using MAXQDA software and conventional content analysis. Validity of the data was approved based on four criteria: credibility, dependability, conformability and transferability.

**Results:**

Five themes emerged through analysis: “unpreparedness to deal with the COVID-19 resulting in disorganized triage”, “threat to the physical and mental health of personnel during the COVID-19 pandemic”, “degradation of the quality of services due to improper triage structure during the COVID-19 pandemic”, “communicating with patients which is neglected during the COVID-19 pandemic” and finally “accountability required to improve the provision of services during the COVID-19 pandemic.

**Conclusion:**

Obstetrics and gynecology service providers and recipients faced formidable challenges in the triage department during this pandemic caused by the complex and ambiguous nature of the Coronavirus. Identifying the problems, barriers and challenges in providing services to patients in this situation especially in triage, can lead to an improvement in the outcome of services.

## Background

The COVID-19 pandemic was recorded as one of the most challenging disasters in the world [1, 2]. The health system needed to adopt a clear policy for dealing with this disease because of its severity. In Iran, according to WHO, from 3 January 2020 to 26 November 2022, there were 7,559,706 confirmed cases of COVID-19 disease with 144,633 deaths (https://covid19.who.int/region/emro/country/ir).

The emergency department is one of the main parts of the health system. Nowadays, the emergency department has become the main site for providing emergency medical care [3, 4]. Research results have shown that during the COVID-19 pandemic, the emergency department was the top priority and the emergency workforce were the first human role players who provided health care services to the patients; accordingly, a heavy workload was imposed on them. Currently, a huge crowd in the emergency department is recorded from diverse countries. Large population of patients admitted to the emergency departments during the COVID-19 pandemic led to the crowdedness in the departments which was aggravated by lack of healthcare personnel and resources, which could have affected the quality of healthcare services [3, 5–8].

A highly sensitive group in the society is the population of pregnant women. Due to their frequent referrals to obstetrics and gynecology ward, it is necessary to provide high quality services in the obstetric triage as it can lead to a higher level of satisfaction and efficiency, as well as the reduction of mortality rate in the emergency departments. Therefore, it is necessary to closely scrutinize the quality of services so that optimally desirable services are provided to clients [8, 9].

Finally, as to our best knowledge, few studies have been conducted to examine this issue, as well as due to the importance of the health services quality in reducing mortality rate and increasing quality of life, this study was conducted with the aim of exploring the obstetric and gynecological service providers’ and recipients’ perception and experience of the services quality in the obstetric triage during the COVID-19 pandemic.

## Methods

This qualitative study was conducted during the COVID-19 pandemic in the obstetric triage ward in the hospitals of Mashhad University of Medical Sciences, Mashhad, Iran, from Sep 2021 to Feb 2022.

Obstetric and gynecological service providers’ and recipients were provided with the necessary explanations about the objectives and method of the study; subsequently, their (Obstetric and gynecological service providers’ and recipients) written consents were obtained.

Since the quality of health care services is a subjective, complex, and multidimensional concept, qualitative research methods were applied in the present study. In addition, the use of these methods was likely to provide deeper insights into the service providers’ and recipients’ perceptions because improving the service quality regardless of the clients’ perceptions, opinions, needs and expectations seems to be less achievable [10–15].

Since the aim of this study was exploration of the perceptions and experiences of the obstetric and gynecological service providers and recipients about the quality of services in obstetric triage, and because there was no similar study in Iran, the conventional content analysis method was used.

The conventional content analysis is commonly applied about a topic when the research literature on that topic is scarce. In the present case, the researchers worked on the study data to create a new viewpoint.

Data collection was performed and continued up to the point of data saturation, i.e. the follow-up questions continued until the interviews did not provide further information and only the previous data were repeated.

After the participants completed the informed consent form, data were collected by in-depth semi-structured interviews. At the interview, the interviewer introduced himself and described the objectives of the study. Besides, he created a friendly atmosphere by asking introductory and warm-up questions (to elicit the demographic information) and forming an appropriate relationship to gain the women’s trust. At the end of the interview, the participants were asked to add any further comments and were informed about further interviews, if any. Each interview session lasted between 45 and 90 min.

The interviews were recorded and transcribed verbatim with the interviewees’ consent. Inclusion criteria for obstetrics and gynecology service providers and recipients in obstetric triage ward included being able to understand and speak Persian in order to express their feelings and experiences, and being interested in participating in the study to talk about the experiences and concerns with the researcher. In addition, an inclusion criterion for obstetrics and gynecology services providers was having at least a 6-month experience of working in an obstetric triage ward during the COVID-19 pandemic. The only exclusion criterion was the participants’ unwillingness to continue cooperating at any stage of the interview.

Triage services in Iran are given to all pregnant women regardless of their gestation status. It includes patients who show early pregnancy symptoms such as threatened miscarriage.

ESI triage system is used in Iran. ESI triage system (Emergency Severity Index) is a five-level system in which, in addition to the severity of the disease and the degree of deterioration of the patient’s clinical condition (acuity), the amount of facilities required by the patient (resource) in the emergency room is also determined. The severity of the disease with the presence or Absence of life or limb threat and presence of danger signs as well as determined vital signs and required facilities are determined based on experience and comparison with similar cases.

Some interview questions for the service providers were: What was the effect of Covid-19 on the provision of obstetric services? What challenges and problems did you face on that occasion? How did these challenges affect your workflow?

Some interview questions for the service recipients were: What problems did you face in the obstetric triage?

During the interviews, the participants were asked to talk about their caregiving experiences in detail. In addition, to guide the interview and in response to the participants’ answers, other questions were asked; e.g. can you explain more in this regard? Have you had any experience in this field that you can explain to me?

During the study process, the interviews were conducted based on the created questions and the researchers directed the questions based on the important created categories.

The study was analyzed based on the principles of conventional content analysis, which includes the following steps: transcribing the entire interview verbatim immediately after each interview, reading the whole text for a general understanding of its content, identifying the conceptual units and initial codes, classifying similar initial codes into more comprehensive categories, and ultimately determining the theme of the categories.

All interviews were recorded, typed word by word, and edited for correspondence with the participants’ verbal protocols. In order to extract the codes, the data were read word by word. With the help of reduction and text compression process, the initial codes were extracted and were then compared based on their similarities and differences and placed in sub-categories. After organizing, the sub-categories were placed in the related categories based on the relationship between them. Finally, after analyzing, interpreting and abstracting the categories, the theme which was more condensed was extracted [16].

The credibility, dependability, conformability and transferability criteria, expressed by Lincholn and Guba, were used to confirm the study findings. Certain measures were taken including spending enough time for data collection and analyses to deeply understand the participants, reviewing the interviews and codes by the research team, building a good rapport with the participants to conduct more in-depth interviews, processing data in the long term, reading the interviews several times to modify the codes, reviewing various stages of analysis in repeated sessions by the research team, and reviewing the codes by other colleagues. Interviews, codes and extracted categories were given to some experts familiar with qualitative research methods; they were asked to review the coding and analysis methods and provide comments on its accuracy. In controversial cases, discussions were made and consensus was achieved. Different stages of analysis were recorded and described to be used for evaluation by external experts [17, 18]. Data were analyzed using MaxQda12 software.

## Results

In this qualitative study, 57 in-depth interviews were conducted with service recipients (24 patients) and service providers (33 triage staff) in the obstetric triage of hospitals affiliated to Mashhad University of Medical Sciences, Iran. Service recipients (age range from 18 to 42 years) were either housewives or employed, and had different levels of education (ranging from elementary school to postgraduate) at a gestational age ranging from 5 to 42 weeks. Service providers (age range from 24 to 56 years) were midwives, obstetrics and gynecology residents, and triage ancillary workers. They had different levels of education ranging from high school diploma to PhD degrees, with working experience from 1 to 28 years (Tables 1 and 2).

**Table 1:** The profile of obstetric and gynecological service providers

**Variable**	**Number (percent)**
Occupation	
Midwife	20(60.6)
OB/GYN resident	6(18.2)
Triage ancillary workers	7(21.2)
**Variable**	**Mean ± SD**
Age(year)	36.939 ± 9.727
Years of working experience	12.151 ± 8.031

**Table 2:** The profile of obstetric and gynecological service recipients

**Variable**	**Number (percent)**
Education level	
Under diploma	7(29.2)
Diploma	13(54.2)
Higher than diploma	4(16.7)
Occupation	
House holder	15(62.5)
Employed	9(37.5)
**Variable**	**Mean ± SD**
Age(year)	27.958 ± 8.002
Gestational age (week)	31.750 ± 1.039

Qualitative analysis of the data led to the emergence of 674 initial codes, 200 sub-sub-categories, 55 sub-categories, 15 main categories and 5 themes (Table 3).

**Table 3:** Main theme, categories and subcategories emerged from data analysis

sub-categories	categories	Themes
limitation on admission due to improper referral of patients to COVID-19 centers	Not knowing how to deal with patient admission at the beginning of COVID-19 pandemic	unpreparedness to deal with the COVID-19 results in disorganized triage
Increased referral of patients in COVID-19 pandemic		
Obligatory regulation on admission of COVID-19 patients in COVID-19 or non-COVID-19 centers		
Improper distribution of patients in non-COVID-19 centers		
Patients’ stress due to improper conditions caused by crowds	Challenge of patient admission and care due to lack of a clear structure	
Delay in investigation of COVID-19 patients due to lack of a specific structure		
Not knowing how to deal with patient admission at the beginning of COVID-19 pandemic		
Waste of time following the confusion of referrals to triage ward		
Concern about transmitting the disease to others, especially the family members	Fear of being infected, the cause of social and family isolation	Threats to the physical and mental health of personnel during COVID-19 pandemic
Social isolation because people thought the medical staff were the carrier of COVID-19		
Concerns about contracting COVID-19		
Challenges of triage personnel infected with COVID-19		
heavy workload due to COVID-19 pandemic	Physical, psychological, and social consequences of COVID-19	
Physical exhaustion due to COVID-19 pandemic		
Mental exhaustion due to COVID-19 pandemic		
Increase in the number of COVID-19 patients due to improper patient admission	Physical and mental exhaustion of patients and staff at the peak of COVID-19	
High-risk pregnancy due to COVID-19 contraction of mother		
Clinical errors at the peak of COVID-19		
Physical exhaustion of personnel at the peak of COVID-19		
Long-term hospitalization of patients suspected to be infected by COVID-19		
Reluctance of patients to refer to COVID-19 centers	Fear of being hospitalized in COVID-19 centers is a factor of hiding the truth	
Fear of being hospitalized in COVID-19 centers is a factor of hiding the truth		
An increase in the number of infection patient due to fear of stigma of COVID-19	Quality of care which is neglected at the time of COVID-19	
Neglect of inpatient care due to unnecessary registration system		
Increase in clinical errors due to the crowd in triage ward		
Neglect of inpatient care due to the crowd in triage ward during COVID-19 pandemic		
Degrading the quality of care due to fear of contracting COVID-19		
Problems or challenges of not having a fixed resident in triage ward	Fast diagnosis due to the presence of a fixed obstetric resident in triage ward	Reduction in the quality of service due to improper triage structure in COVID-19 pandemic
Benefits of having a fixed gynecologist resident in triage ward		
Negligence in following health protocols	Necessity of and personnel cooperation in systematically observing COVID-19 conditions in triage section	
The need for regulations of following the health protocols		
Adequate care in proper triage ward		
The requirement for a purposeful structure of tasks division in the triage ward		
Dissatisfaction due to not following the compliance plan	Dissatisfaction with lack of proper triage infrastructure in COVID-19 conditions	
Patient’s unreasonable expectations of triage services during COVID-19 pandemic		
Lack of space due to high patient referrals		
Challenges of triage equipment shortage		
The benefits of creating an outpatient room which is monitored regularly in triage ward		
Challenges of not having an isolation room prepared for COVID-19 patients		
An increase in the cost of treatment despite of being a public hospital	Deficiency in purposeful and efficient triage management in COVID-19 conditions	
The need to combine clinical education with treatment in triage training centers		
Imposing a cost on the health system due to COVID-19 pandemic		
Low level of job satisfaction due to not receiving the payment assigned for health care staff in COVID-19 pandemic		
Lack of enough motivation to work due to unfair payment assigned for health care staff in COVID-19 pandemic		
Patient’s comfort following empathetic communication	Empathetic communication: giving care compassionately in triage ward	Communicating with patients which is neglected at the time of COVID-19
The obligation for effective communication between the service provider and the recipient		
Improper communication with family of the patient	Fear of being infected, the cause of poor communication between patients and staff	
Improper relationship due to fear of disease transmission		
Commitment and effort of the staff in the difficult conditions of COVID-19 pandemic	Commitment to work in difficult COVID-19 conditions	Accountability required to improve the provision of services during the COVID-19 pandemic
Patient’s satisfaction with the responsibility of personnel		
Effective care as the result of team work	Effective care requirements	
Feeling of power as a result of experience and skill in performance		

The themes were 1- “ unpreparedness to deal with the COVID-19 outcomes in disorganized triage”, 2- “threat to the physical and mental health of the personnel during the COVID-19 pandemic”, 3- “degradation of the quality of service due to the improper triage structure during the COVID-19 pandemic”, 4- “communicating with patients which was neglected during COVID-19 pandemic” and 5- “accountability required to improve the provision of services during the COVID-19 pandemic”.

### Unpreparedness to deal with the COVID-19 outcomes in disorganized triage

This concept was extracted from two main categories: “Not knowing how to deal with patient admission at the beginning of the COVID-19 pandemic” and “the challenge of patient admission and care due to lack of a clear structure”.

These categories indicate factors such as the requirement of COVID-19 patient admission that increased the disorganized referral of patients and improper distribution of patients in non- COVID-19 centers. These factors put negative effect on the quality of obstetric service in the triage ward during COVID-19 pandemic.

“We are legally obliged to admit every COVID-19 patient who is referred to the hospital, although this ward is a non-COVID-19 center. Unless we can really admit the patient and the patient has a non-emergency condition, we are allowed to refer that person elsewhere” (health care service provider).

The lack of clarity in the referral process of COVID-19 patients during the early days of this crisis perplexed the healthcare staff about deciding whether to refer or admit the patient, or even refer patients to the triage ward.

“Patients had to be admitted to the COVID-19 emergency room, but they did not know it. Neither did we know whether to refer the patient to the COVID-19 emergency or not, since there was no a specific protocol about this new disease” (health care service provider).

Confusion about referrals in the triage ward wasted a lot of time, subsequently, it put a bad effect on the quality of care and services.

“A patient with COVID-19 disease was referred to our hospital from a doctor’s office. Our hospital is a non- COVID-19 center so the patient had to be referred to a COVID-19 center instead. Finally, the patient was referred to a COVID-19 center. Wrong patient referrals generally took up our valuable time for other patients” (health care service provider).

### Threats to the physical and mental health of personnel during the COVID-19 pandemic

This concept is extracted from 5 main categories: 1- “Fear of being infected, the cause of social and family isolation”, 2- “Physical, psychological, and social consequences of the COVID-19 disease”, 3- “Physical and mental exhaustion of patients and staff at the peak of the COVID-19 pandemic”, 4- “Fear of being hospitalized in COVID-19 centers is a factor of hiding the truth” and 5- “Quality of care which is neglected during the COVID-19 pandemic”. This concept represents the factors that affect the quality of services provided in the obstetric triage and its consequences.

The crowded triage wards and the heavy workload, especially at the peak of the COVID-19 pandemic, reduced the physical strength of the personnel.

“We do not rest at all at night because we have to take care of 40-50 patients in each shift. Especially at the peak of the COVID-19 pandemic, we become too exhausted after each shift in a way that we cannot even do our daily routines” (health care service provider).

This heavy workload in triage ward has also negatively affected the family and emotional relationships of the staff.

“We have several work shifts in a week. We do not have time to spend with our family and take care of our children at all. We cannot support our children emotionally and pay attention to their nutrition and education” (health care service provider).

Fear of being hospitalized in a COVID-19 center, fear of the COVID-19 stigma and being rejected or fear of being rejected caused the patients to hide the truth of their being infected with the COVID-19 disease, which leads to further transmission of the disease to others, especially to the triage personnel.

“Many patients do not say they are infected but when we check the system, we see that the patient has a positive PCR. Because they are afraid of being hospitalized in a COVID-19 center” (health care service provider).

If the triage personnel are infected with COVID-19, we will face a lot of problems such as absence of the personnel due to sick leave, which imposes heavier workload on other personnel.

“The staff is too exhausted now. Staff who is infected with the COVID-19 disease took a sick leave so it imposes heavier workload on other personnel. We don’t even have a day off” (health care service provider).

Fear of encountering the COVID-19 patients and the possibility of being infected with the virus can have a negative effect on the quality of patient care in the triage ward.

“As soon as a patient says, ‘my PCR is positive’, I subconsciously take the patient away since I do not feel safe although it is our duty to examine the patient” (health care service provider).

### Reduction in the quality of service due to improper triage structure during the COVID-19 pandemic

This concept is extracted from 4 themes: 1- “Fast diagnosis due to the presence of a fixed obstetric resident in the triage ward “, 2- “Necessity of personnel cooperation in systematically observing the COVID-19 conditions in the triage section”, 3- “Dissatisfaction with lack of proper triage infrastructure under COVID-19 conditions” and 4- “Deficiency in purposeful and efficient triage management under COVID-19 conditions”. This concept represents the factors that affect the quality of structure criteria in the obstetric triage. Lack of a fixed resident in the triage ward is one of the challenges of obstetric triage especially under COVID-19 conditions which causes problems such as delayed patient care, long patient stay in the emergency ward, patient dissatisfaction and even neglecting the emergency patient.

“Under these conditions, we should have fixed residents in the COVID-19 center. It takes a long time to decide about a patient because we have to wait for a resident to return to the center. These factors cause the patient to be dissatisfied with the service provider.

“We must have a fixed emergency resident in the COVID-19 center. The patient may need immediate medical care while I am visiting patients in other wards” (health care service provider).

Lack of an isolation room for COVID-19 patients is another challenge in obstetric triage ward, which increases the risk of transmitting this disease to other patients and staff.”

“There is no isolated room for PCR-positive patients in the triage ward. Several pregnant women are visited at the same time, one of whom may be infected with COVID-19, so the disease transmits to others quickly. It is really important to isolate COVID-19 patients from others in the emergency ward” (health care service provider).

Despite the obligation of admitting COVID-19 patients in all centers and also heavy workload of the healthcare staff, the payment assigned for health care staff did not include the personnel of non- COVID-19 centers. This unfairness put extra negative effect on providers’ motivation in caring for patient.

“We have a heavy workload. There is no substitute for those care providers who left the ward. We have to admit the COVID-19 patients but the officials say the extra payment, which is assigned for health care staff during the COVID-19 pandemic, does not include the staff who work in non- COVID-19 centers” (health care service provider).

### Communicating with patients who are neglected during the COVID-19 pandemic

This concept is extracted from two main categories: “Empathetic communication: giving care compassionately in the triage ward”, “Fear of being infected, the cause of poor communication between patients and staff”; it indicates the need for effective communication between the health care providers and the recipients because empathetic communication with the patients helps them calms down. However, in most cases, due to the fear of being infected, the staff are unable to communicate properly with patients and those who accompany them. This negatively affects the quality of services provided in the obstetric triage ward.

“Before this pandemic, we communicated very easily with the patient, we did not wear a mask so the patient could see our smiles and our communication with the patient was much better but now our relationship with the patients is not as friendly” (health care service provider)

The COVID-19 conditions leads to patients’ and their families’ dissatisfaction, and inappropriate behavior of the staff.

“I was in so much pain and in the hallway, there was only one bed on which I was lying. The staff said, do not sleep, and get up. Their behavior was not good. I said why you treat me like this, they said if you wish, you may go to another hospital” (health care service recipient).

“Do not come to the room, do not stand behind the door, and do not talk. I felt bad to be treated like this so I complained about that but they said that you could leave the hospital” (health care service recipient).

### Accountability required to improve the provision of services during the COVID-19 pandemic

This concept is extracted from two main categories: “Commitment to work in difficult COVID-19 conditions” and “Effective care requirements”. The commitment and effort of the triage staff under these difficult conditions had led to the recipients’ satisfaction with triage services, and affected quality of structure criteria provided in the obstetric triage ward.

“They examined me. Everything was fine. I felt good. Their behavior and accountability were good. Despite COVID-19 conditions, when the emergency room was overcrowded, everything was better than I expected” (health care service recipient).

The staff normally endure the difficulty of COVID-19 conditions and establish a proper relationship with the patient and their family.

“All patients are stressed during this pandemic and their tolerance is low. If there is a problem, I will try to avoid conflict with patients and their family” (health care service provider).

Personnel in the triage ward are committed to do their duties despite the challenges such as the lack of personal protective equipment.

“Because we are not provided with personal protective equipment such as the N95 mask, I get stressed about being infected as a result of giving care to COVID-19 patients. But it does not affect my work; I do take my own responsibilities” (health care service provider).

On the other hand, team work among healthcare personnel, especially under COVID-19 conditions, affects the provision of optimal care to patients and effectiveness of services provided in the triage ward.

“When a patient is admitted, I cannot give them all required services. There must be a teamwork to provide better services. It is really a process. A resident must take a complete patient history and then send it to me in order to transfer the patient. Facing a problem in any step of this process causes trouble” (health care service provider).

The experience and skills of the caregiving staff are the most effective factors in the triage ward, because it exerts remarkable effect on providing optimal care and accelerating the patient assignment, especially under emergency situations.

A health care service provider said:”Skilled and experienced midwives can correctly and quickly distinguish between an emergency patient and a non-emergency one just by taking a brief look at patient’s complexion “(health care service provider).

## Discussion

The emergence of the COVID-19 disease turned into a major global health challenge, and all health policymakers made efforts to reduce its mortality and morbidity. This qualitative study was conducted to explain the obstetric and gynecological service providers’ and recipients’ perception and experience of the quality of services in the obstetric triage ward during the COVID-19 pandemic to have a better management of patients and possible control of the disease in this ward.

One of the concepts in the present study was “unpreparedness to deal with COVID-19 outcome in disorganized triage”. The emergence of the disease, the varying behaviors of the Coronavirus, different symptoms and course of the disease perplexed the medical staff and caused fear and anxiety among patients [19, 20]. In addition, the ambiguous nature of the Coronavirus and the unknown process of the disease have caused psychological disorders in different population groups [21]. At the beginning of the pandemic, the unknown nature of the virus sometimes made medical staff afraid of being infected with the virus and transmitting the disease to their own family members. As the pandemic continued, the medical staff became more competent in overcoming their fear. Despite their fatigue, the staff attempted more than ever to take care of COVID-19 patients while taking the burden of wearing uncomfortable protective uniforms during long shifts of care giving [22].

The long triage process of COVID-19 patients due to lack of a clear structure affected the quality of services. A study by Paul et al. showed that the management of patients care in triage centers positively affected patients’ satisfaction and reduced the duration of patient’s stay in the triage ward [23].

One of the emerging concepts in the present study was “threat to the physical and mental health of personnel under COVID_19 conditions”. This issue is more common in patients referring to medical centers, because of their fear and anxiety of being hospitalized in the COVID_19 center; therefore, they tend to hide the reality of being infected with the COVID-19 disease. Also, psychological factors are among the most dangerous ones when dealing with crises and disasters. For instance, “fear of death” implies an emotional response to the perception of real or imaginary symptoms along with threats to survival [24]; it can also compromise patients’ immune system [25]. In a phenomenological study, based on the experience of COVID-19 patients, “fear or anxiety of death” was extracted as one of the main themes [26]. In other studies, “fear of death” has been identified as a serious psychological consequence among patients with COVID_19 disease and their caregivers [27, 28]. Patients with the COVID_19 disease tend to get anxious and worried due to the ambiguity of the nature of the disease. Meanwhile, nurses do not have the required experience and skills to deal with these patients’ anxiety [29]; even they are not familiar with the term “Corona phobia” or fear of Corona. These factors contribute to patients’ further anxiety. Therefore, it is essential to strengthen midwives’ coping strategies by which they become more competent to deal with this psychological disorder [30]. The triage healthcare workers are at risk of physical and mental consequences directly as the result of providing care to patients with COVID-19. Result expressed implementation strategies to reduce the chances of infections, shorter shift lengths, and mechanisms for mental health support could reduce the morbidity and mortality amongst them [31].

Another concept in the present study was “reduction of service quality due to inappropriate triage structure during the COVID-19 pandemic”. A study by Cavallaro et al. showed that one of the reasons for providing delayed emergency services was the lack of professionally qualified staff [32]. The results of the present study showed that under the condition of COVID-19 pandemic, many of the personnel in the triage ward were themselves infected with the virus. Another study by Andersson et al. showed that skills, competence as well as presence of the personnel in the triage ward were important factors in terms of correct decision-making about patients admitted to the triage ward [33]. Consequently, when a pregnant mother is properly and successfully examined by an experienced midwife for referral purposes or for discharging from hospital, then the clients’ satisfaction will definitely increase due to fast diagnosis [34]. The results of the present study showed that due to the lack of a fixed resident in the COVID-19 center, the process of diagnoses took longer in the obstetric triage; therefore, it led to pregnant mothers’ dissatisfaction, especially under the COVID_19 conditions. The findings of a study by Rashidi et al. showed that one of the causes of pregnant mothers’ dissatisfaction originated in long waiting time for caregiving and patient assignment [35]. The results of another study by Johnson et al. also showed that long waiting time led to patients’ leaving without initial examination, delay in receiving the necessary care and treatment, dissatisfaction, and an increase in mortality rate in some cases [36].

Another concept in the present study is “communication with the patient which is neglected under COVID_19 conditions”. Communication is essential for obtaining an accurate history and collecting necessary information to make the right diagnosis [37]. The results of other studies have shown that proper communication between health care service providers and recipients was a significant factor in providing effective and dynamic care [38]. According to these studies, the heath care service recipients expect to be appropriately treated by heath care providers and receive good quality of care during the triage process [39]. Establishing relationships for triage is necessary to assess the clinical situation. The assessment includes subcategories such as general condition, time factor, threats, pain, parameter, physical examination and an overview, the result of which indicates prioritization [33].

In the research Rashidi-Fakari with title “The Quality of the Maternity Triage Process: a Qualitative Study” concluded that most participants identified care as the main factor in the quality of the delivery triage process. Standardization and adaptation of procedures and criteria in care in both examination and history taking are effective in the quality of the process of providing maternity triage services. Incorrect initial assessments, especially during triage, have been reported to increase errors and cause complications and complications [40].

Another concept in the present study is the following: “Accountability is required to improve the provision of service under the condition of COVID-19 pandemic”. Accountability is one of the main goals considered by policy makers and health systems managers. According to the World Health Organization report in 2000, one of the three fundamental goals of health systems is to meet the patients’ needs [41], because any system must attempt to meet the legitimate needs of its service recipients in order to be successful. Accountability has recently gained an increasing significance since it is associated with patient satisfaction. Additionally, improving accountability can increase the sense of comfort and better cooperation between health care service recipients and providers; therefore, it is expected to lead to the ever-increasing health of the society. Health care providers in the obstetric triage would further feel the need to meet pregnant women’s needs as they are considered as a highly susceptible group. Especially during the COVID-19 pandemic, it is more important to provide pregnant women with high quality health care services to achieve greater satisfaction [42–44].

During the COVID-19 pandemic, one of the most important factors was the pregnant women’s fear and anxiety when presenting to the obstetric triage. According to previous research, fear and stress cause the secretion of oxytocin that leads to the tendency to communicate with others [45], so the appropriate accountability triage personnel play an important role in the pregnant mothers’ sense of convenience and comfort. Commitment to work and effective interaction between service providers and recipients lead to the development of a more accountable health system [46, 47].

Important infrastructure measures are required to improve the situation during the conditions of this crisis [42].

Since the COVID-19 disease may be considered as the most devastating contemporary global challenge and threat, meeting the needs of the service recipients, especially the pregnant mothers, is essential. The findings indicate that interactions and communication were identified as a main factor in the quality of the delivery triage process, especially in the conditions of Covid-19. Accuracy and speed of operation are very important for triage and critical situations. Because carelessness and errors in triage have a negative effect on clients and the next stages of care. For these purposes, we should plan and take timely and appropriate measures to control and reduce the consequences of this disease.

In order to improve the quality of the caregiving process such as early diagnosis, treatment, hospitalization and recovery of patients, the following may be considered: the formation of a crisis team, preparing and compiling guidelines and protocols, and screening all households through designing an effective health system.

Also, the following measures may be recommended: taking measures to recruit further personnel, using new infrastructure and optimal use of resources, employing a permanent resident in the triage ward on a full-time basis, revising the principles of triage for infected patients, and assigning hospitals as COVID-19 centers in each city for the referral of infected and suspected patients in crisis COVID-19.

## Conclusion

The present study showed that obstetric and gynecological service providers and recipients in medical centers faced increasing challenges and were stressed during the COVID-19 pandemic.

The main concern of the participants in the present study was the fear of contracting Covid-19 and likely threats to their pregnancy. This concern is caused by the complex, ambiguous and unknown nature of the corona virus. Therefore, women with the Covid-19 disease experienced conflicts of fear and anxiety since their symptoms continued to worsen. At the community level, it seems that ignorance of symptoms, delay in the referral, resistance against hospitalization, preference of home quarantine despite the exacerbation of symptoms, and consequently a delay in providing effective care of pregnant patients are among the issues that require immediate attention.

The present study on the experiences of obstetric service providers and recipients ultimately led to the identification of problems, barriers and challenges in providing services to pregnant mothers during this pandemic. Moreover, improving the quality of midwifery triages under crisis conditions turns to a serious goal for policy makers.Therefore, we are now able to contemplate on the existing problems and barriers in the obstetric triage to plan for healthier mother and newborns.

## Data Availability

The datasets used and/or analyzed during the current study are available from the corresponding author on reasonable request.
